# AZ91 Magnesium Alloy CMT Cladding Layer Processed Using Friction Stir Processing: Effect of Traverse Speed on Microstructure and Mechanical Properties

**DOI:** 10.3390/ma17102348

**Published:** 2024-05-15

**Authors:** Huichao Zhao, Junqi Shen, Shengsun Hu, Yahui Zhen, Yang Chen

**Affiliations:** 1 Tianjin Key Laboratory of Advanced Joining Technology, Tianjin University, Tianjin 300354, China; zhao4672262@gmail.com (H.Z.); huss@tju.edu.cn (S.H.); 15102220135@163.com (Y.Z.); chenyang236@tju.edu.cn (Y.C.); 2 School of Materials Science and Engineering, Tianjin University, Tianjin 300354, China

**Keywords:** magnesium alloy, CMT cladding, FSP, traverse speed, microstructure, mechanical properties

## Abstract

Friction stir processing (FSP) is a solid-state treating method to enhance the mechanical properties of materials by altering their microstructure. In this study, FSP was applied to the AZ91 magnesium alloy cladding layer prepared using cold metal transition (CMT) technology, and the purpose was to investigate the effect of the traverse speed of the H13 steel stirring head under a constant rotation speed on the microstructure and mechanical properties of the cladding layer. The results demonstrated that FSP could effectively decrease the grain size of the cladding layer and lead to the dispersion and dissolution of the coarse β-Mg_17_Al_12_ second phase into the α-Mg matrix. The mechanical characteristics of the processed cladding layer were significantly enhanced compared to the unprocessed cladding layer due to the grain refinement and second-phase strengthening induced by FSP. When the stirring head rotation speed was set at 300 r/min, the average microhardness and tensile properties of the specimens showed a tendency of initially increasing and then dropping as the traverse speed increased. The cladding layer, obtained at a traverse speed of 60 mm/min, displayed optimal mechanical properties with an average microhardness, tensile strength, and elongation of 85.6 HV_0.1_, 278.5 MPa, and 13.4%, respectively.

## 1. Introduction

Magnesium alloys, being the most lightweight structural metallic materials used in engineering, possess exceptional physicochemical characteristics including low density, high specific stiffness and specific strength, recyclability, and effective damping properties [1,2,3,4]. Additionally, magnesium is widely available in Earth’s crust, ranking as the eighth most abundant element on Earth [5]. In today’s world, there is a growing trend towards structural lightweighting. Magnesium alloys, due to their aforementioned advantages, have been shown to be highly valuable in several industries such as automotive [6,7,8], aerospace [9], biomedical [10,11], and national defense [12]. Nevertheless, the comparatively low modulus of elasticity, inadequate wear resistance, and elevated corrosion rate of magnesium alloys make their surfaces prone to wear, scratches, and other forms of damage when in use [13]. Cold metal transfer (CMT)-based surfacing technology presents a viable way to repair the damaged structural components made of magnesium alloy. The low thermal input and stable welding process of this technology are its distinguishing features [14]. However, the surface repairing process of magnesium alloys through the use of the surfacing welding technique frequently results in the formation of defects like inclusions, porosity, and cracks [15]. Therefore, it is imperative to introduce an appropriate post-treatment technique to improve the performance of the repaired parts.

The current primary methods employed for altering the surface properties of magnesium alloys include the physical vapor deposition technique, chemical vapor deposition technique, laser surface modification technique, and FSP technique [16]. FSP is derived from friction stir welding (FSW) and is primarily employed for the purpose of altering the microstructure and properties of materials. The primary components of the FSP tool are the stirring head consisting of a pin and a shoulder. The high-speed rotating shaft shoulder comes into direct contact with the material surface during processing, resulting in significant heat generation that softens the metal material. Simultaneously, the rapid stirring motion of the pin causes the material to undergo substantial plastic deformation. The FSP can homogenize and densify the microstructure of the material as well as refine the grains, resulting in the improvement of material properties [17,18,19]. FSP has the following advantages over other methods. Firstly, FSP does not produce harmful gases and noise, and it is a green and pollution-free method. Secondly, FSP can carry out high-speed and high-efficiency processing. Thirdly, the stirring head shape, rotational speed, and traverse speed can be adjusted easily to suit the material. Lastly, FSP does not require additional heating equipment, and it is easy to operate.

Both domestic and international researchers have conducted numerous investigations on the FSP modification of Mg alloy materials and have obtained significant research outcomes. Rokkala et al. [20] showed that, after FSP, the grain size of the as-cast Mg–Zn–Dy alloy was reduced from 60 μm to 3 μm, and the hardness, yield strength, tensile strength, and elongation of the alloy were increased by 46%, 60%, 55%, and 53%, respectively. Elyasi et al. [21] utilized the FSP technique to treat a Mg–Zn–Si alloy, and they observed that the mechanical properties of the alloy were enhanced as a result of FSP-induced grain refinement and the reduction in porosity content. Sharahi et al. [22] found that the yielding strength, tensile strength, elongation, and energy absorption of the as-cast AZ91 Mg alloy were significantly increased by 42%, 80%, 350%, and 830%, respectively, due to the refinement of the grains and dissolution and dispersion of the Mg_17_Al_12_ intermetallic phases induced by the FSP process, compared to the unprocessed sample.

The key determinants of the quality of a friction stir processed sample are the rotational speed and traverse speed of the stirring head. These two parameters directly influence the amount of heat generated and the material deformation throughout the FSP process [23]. Kondaiah et al. [24] found that the rotating speed of the stirring head during FSP of ZE41 Mg alloy had an impact on the degree of refined grains and the breadth of the stirred zone (SZ). Girish and Anandakrishnan [25] found that a high traverse speed above 70 mm/min during FSP of AA7075 Al alloy could lead to insufficient heat input, thus decreasing the plastic deformation of the material. John et al. [26] demonstrated that an increase in the rotation speed or traverse speed of the stirring head in a proper range could lead to a rise in the tensile strength of AA 2014-T6 Al alloy, and the interaction between the traverse speed and rotation speed had a substantial impact on both the microstructure and microhardness of the SZ. Luo et al. [27] reported that the grain size and the proportion of large angular grain boundaries of the AZ61 Mg alloy exhibited an increase as the ratio of the rotation speed to the traverse speed increased. Hu et al. [28] FSP-treated ZK60 magnesium alloy plates at various rotational and traverse speeds. The results show that fine recrystallized grains and a high-angle grain boundary can be observed at different process parameters. The grain size and the proportion of high-angle grain boundary increase as the rotational speed increases, whereas the grain size reduces and the proportion of high-angle grain boundary increases as the traverse speed increases.

In summary, changes in the rotating speed and the traverse speed of the stirring head have a significant impact on the microstructure and mechanical properties of the processed materials. However, much of the research undertaken on the effects of FSP parameters on the properties of magnesium alloys has mostly focused on cast magnesium alloys. The issues experienced by the cladding layer manufactured using additive manufacturing techniques are distinct from those encountered by cast alloys. The main challenges encountered by the cladding layer can be succinctly described as follows: mechanical property anisotropy caused by the uneven distribution of microstructure, thermal stresses and deformation resulting from uneven heating and cooling during the additive manufacturing process, and pore defects arising from insufficient fusion between layers [29]. Investigating the influence of FSP parameters on the fusion cladding layer of magnesium alloy would help to address the mentioned difficulties, hence greatly broadening the potential uses of magnesium alloys.

Hence, the objective of this study was to examine the impact of the traverse speed of the stirring head on the microstructure and mechanical characteristics of the AZ91 magnesium alloy CMT cladding layer subjected to FSP. Furthermore, a comprehensive examination was carried out on the macroscopic characteristics, crystalline grain size, morphology, and distribution of the secondary phase, as well as the mechanical characteristics of the cladding layer achieved at different traverse speeds.

## 2. Experimental Procedure

An as-cast AZ91 magnesium alloy plate was used as the substrate, and the dimension of the substrate was 250 mm × 150 mm × 6 mm. The selected cladding material for this investigation was AZ91 magnesium alloy welding wire, which had a diameter of 1.2 mm. Table 1 presents the chemical components of both the substrate and the cladding wire.

The CMT cladding system consisted of a Motoman HP6 six-axis robot and a Fronius CMT Advanced 4000R welding machine. For the CMT cladding process, the used shielding gas was pure argon, and the specific cladding parameters can be found in Table 2.

**Figure 1:**
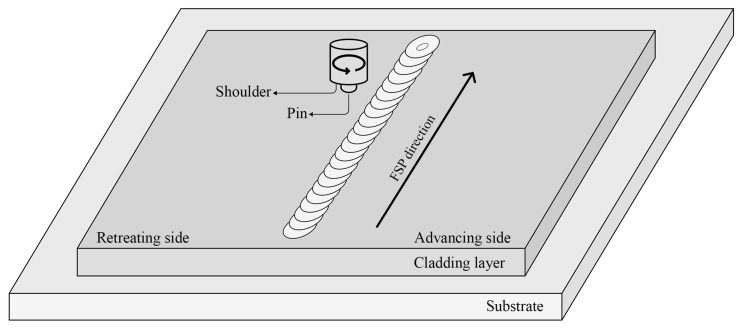
Schematic diagram of the FSP treatment.

AZ91 magnesium alloy CMT arc cladding layers were processed by using a gantry-type FSP machine (HT-JM16×15/2). A conical threaded stirring head made of H13 steel with a shoulder diameter of 15 mm and a pin length of 5 mm was used. The pin had a tip diameter of 4 mm and a root diameter of 6 mm. As shown in Figure 1, the processing direction was aligned with the travelling direction of the welding torch during the CMT cladding process. The FSP parameters are indicated in Table 3. During the FSP of the AZ91 magnesium alloy cladding layer, the rotation speed of the stirring head was set at 300 r/min. The tests were then conducted at traverse speeds of 30 mm/min, 60 mm/min, 90 mm/min, and 120 mm/min, respectively. Subsequently, the samples acquired at various traverse speeds underwent microstructure identification and mechanical characteristic tests. The impact of the traverse speed of the stirring head on the cladding layer was examined by analyzing the collected data.

The metallographic samples, which were cut perpendicular to the processing direction (i.e., the direction in which the welding torch was moving), were taken from the cladding layer before and after FSP. These samples were polished and then etched using a solution containing 10 mL of hydrochloric acid and 100 mL of alcohol. The cross-sectional macroscopic morphology of the SZ was examined using a super depth-of-field microscope (ZEISS Smart Zoom 5), and the microstructure of the samples was analyzed by using an optical microscope (ZEISS VERT A1) and a scanning electron microscope (SEM, JSM-7800F).

**Figure 2:**
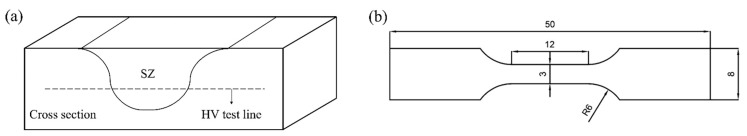
Schematic of the sample for (**a**) microhardness test and (**b**) tensile test.

The microhardness of the unprocessed and treated cladding layer was measured and studied using an HV-1000A Vickers microhardness tester. The applied force was 100 g and the holding period was 15 s. As shown in Figure 2a, the microhardness test line on the cross-sectional sample of the cladding layer after FSP was positioned at a distance of 3 mm from the upper surface of the sample, and the interval between the adjacent test points in the SZ and the other region was 0.5 mm and 1.0 mm, respectively. The dimensions of the tensile sample are shown in Figure 2b. The tensile characteristics of the cladding layer were assessed using an electro-mechanical universal testing equipment (INSTRON 5848) at room temperature, and the loading rate was 0.3 mm/min. Three repetitions for each type of sample were performed, and the average value was considered as the final result. After the tensile test, the fracture morphology was observed and analyzed using a SEM (JSM-7800F).

## 3. Results and Discussion

### 3.1. Macrostructure

**Figure 3:**
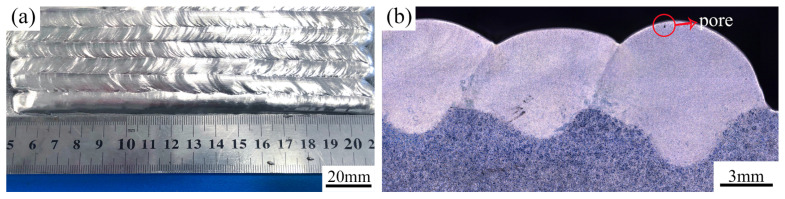
Macromorphology of the cladding layer: (**a**) surface; (**b**) cross section.

**Figure 4:**
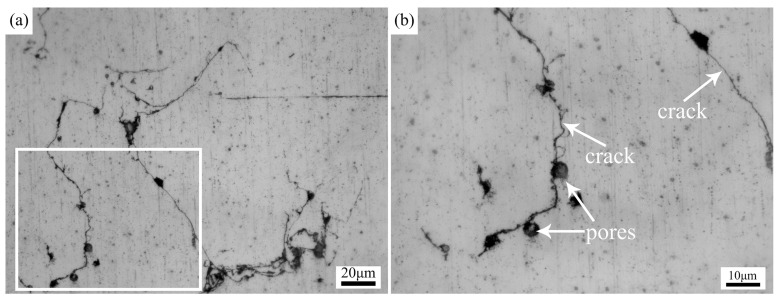
Defects in the cladding layer: (**a**) low magnification; (**b**) high magnification.

As shown in Figure 3, the CMT cladding layer of the AZ91 Mg alloy had a relatively flat and well-formed surface, while some pores could be observed in the top region of the cladding layer. According to the cross-sectional metallographs of the cladding layer, as shown in Figure 4, pores and microcracks were formed in the cladding layer.

By integrating the findings of relevant research [30], it is possible to determine that the pores in the cladding layer are hydrogen pores. Due to the high solubility and low solubility of hydrogen in the liquid and solid magnesium alloy, respectively, some of the hydrogen dissolved in the liquid molten pool became trapped in the cladding layer during the fast solidification process induced by the high thermal conductivity of the magnesium alloy, leading to the formation of hydrogen pores.

**Figure 5:**
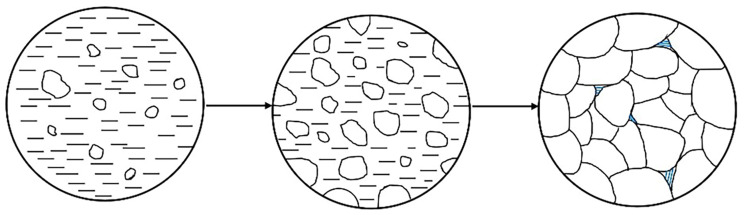
Schematic diagram of liquid film formation during the solidification of magnesium alloy.

Cracks easily occur during the welding process of magnesium alloy, and there are two reasons for crack initiation and propagation. On one hand, due to the high stress concentration around the hydrogen pores, these pores could act as crack initiation sites, leading to the formation of microcracks under tensile stress [31]. On the other hand, as depicted in Figure 5, the grain size gradually increased during the solidification process of magnesium alloy, leading to a reduction in the mobility of the liquid metal of a low-melting-point eutectic, and the liquid metal took the form of a thin film at the grain boundaries during the final stage of solidification. Due to the large linear thermal expansion coefficient of magnesium alloys [32], a significant distortion of the solidified alloy induced by solidification shrinkage occurred, and the liquid film fractured under tensile stress [33]. Therefore, the cracks initiated at pores and propagated along the grain boundaries, as shown in Figure 4.

**Figure 6:**
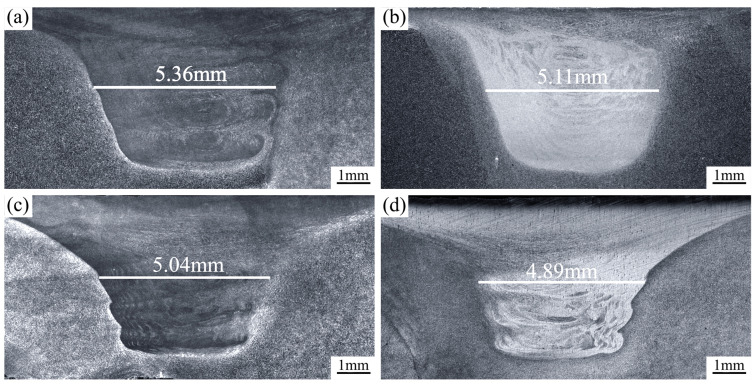
The cross-sectional macroscopic morphology of the SZ at various traverse speeds: (**a**) 30 mm/min; (**b**) 60 mm/min; (**c**) 90 mm/min; and (**d**) 120 mm/min.

Figure 6 illustrates the cross-sectional macroscopic structure of the SZ at different traverse speeds. The SZ had a basin-like shape with a broad top and a narrow bottom for each parameter condition. The SZ exhibited no imperfections, such as pores or cracks. Additionally, when the traverse speed of stirring head was set at 30 mm/min, 60 mm/min, 90 mm/min, and 120 mm/min the intermediate width of the SZ was 5.36 mm, 5.11 mm, 5.04 mm, and 4.89 mm, respectively. Increasing the traverse speed at a constant rotation speed could lead to a decrease in the maximum temperature in the SZ, thus resulting in a decrease in the plastic deformation degree of the material in the SZ. Consequently, the width of the SZ at the intermediate position reduced as the traverse speed of the stirring head increased.

### 3.2. Microstructure

**Figure 7:**
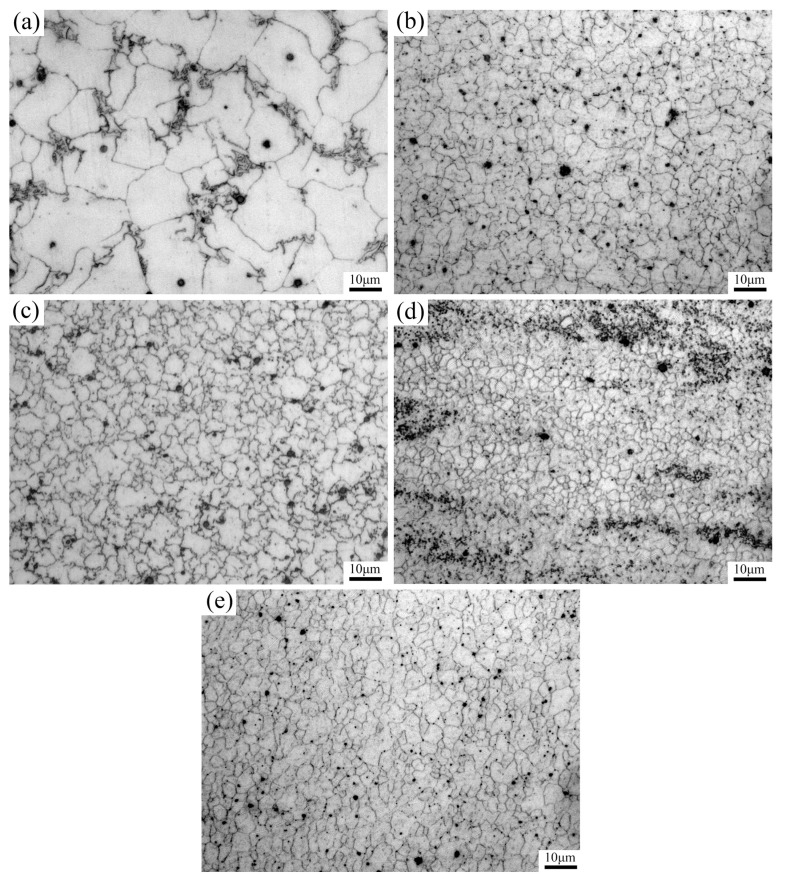
Metallographic microstructure of the SZ at various traverse speeds: (**a**) before FSP; (**b**) 30 mm/min; (**c**) 60 mm/min; (**d**) 90 mm/min; and (**e**) 120 mm/min.

Figure 7a demonstrates that the microstructure of the unprocessed cladding layer closely resembles the that of the welded zone created using gas tungsten arc welding (GTAW), as described in reference [34], and the unprocessed cladding layer consists mainly of coarse equiaxed α-Mg grains and β-Mg_17_Al_12_ secondary phases at the grain boundaries, with an average grain size of approximately 17.6 μm. When the traverse speeds were 30 mm/min, 60 mm/min, 90 mm/min, and 120 mm/min the average grain size of the corresponding SZ was 6.2 μm, 5.6 μm, 3.4 μm, and 6.0 μm, respectively. Compared with the unprocessed cladding layer, the grains of the cladding layer were considerably refined after FSP. Moreover, there was a notable alteration in the morphology and distribution of the second phase, and the coarse Mg_17_Al_12_ phases, which distributed at the grain boundaries before FSP, dispersed into the α-Mg grains with small size after FSP. Under the constant rotation speed condition, an increase in the traverse speed could lead to a decrease in heat input, resulting in a reduction in the grain size of the SZ. Nevertheless, when the traverse speed was raised from 90 mm/min to 120 mm/min, the average grain size of the SZ exhibited a noticeable upward trend. When the traverse speed was excessively high, the plastic deformation of the material in the SZ was inadequate, resulting in uncompleted dynamic recrystallization and a combination of coarse and fine grain regions in the SZ. Therefore, when the traverse speed increased beyond a proper range, an increase rather than a decrease in the average grain size of the SZ occurred.

**Figure 8:**
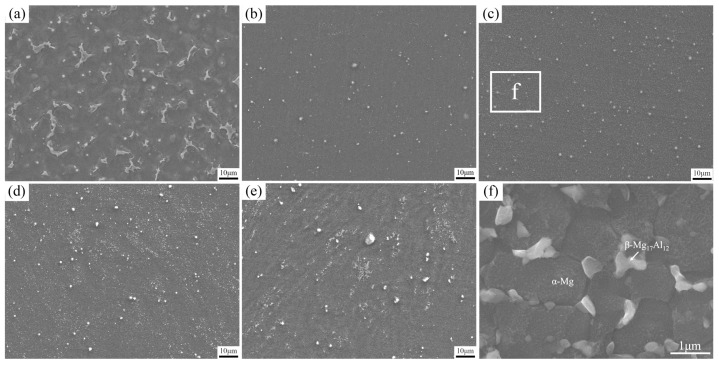
SEM images of the SZ at various traverse speeds: (**a**) before FSP; (**b**) 30 mm/min; (**c**) 60 mm/min; (**d**) 90 mm/min; (**e**) 120 mm/min; and (**f**) magnification of the marked zone in (**c**).

Figure 8a illustrates the presence of several elongated secondary phases of β-Mg_17_Al_12_ scattered along the grain boundaries in the cladding layer before FSP, and the α-Mg matrix was surrounded by the β-Mg_17_Al_12_ phase and the eutectic α-Mg. Based on the phase diagram of the Al–Mg alloy [35], it is evident that the homogeneous crystallization reaction of L→α-Mg occurs when AZ91 magnesium alloy solidifies under equilibrium conditions. As the temperature decreases to the solidus, the crystallization reaction ends and the liquid phase completely transforms into α-Mg solid solution, resulting in the homogenization of the composition through the diffusion of Al atoms. Subsequently, as the temperature further decreases beyond the solidus, the β-Mg_17_Al_12_ phase gradually precipitates around the grain boundaries. Finally, the microstructure of the AZ91 magnesium alloy is composed of α-Mg and β-Mg_17_Al_12_ phases when the temperature reaches room temperature. However, the deposited cladding layer of AZ91 magnesium alloy experienced rapid cooling during the CMT arc cladding process. This caused the deposited alloy to solidify in a non-equilibrium state, preventing the uniform diffusion of Al atoms in the α-Mg solid solution and, thus, leading to Al enrichment in the un-solidified liquid phase. As a result, a eutectic phase was formed via the eutectic reaction [36]. Hence, the cladding layer of AZ91 magnesium alloy contained a portion of the eutectic structure, alongside the α-Mg matrix and β-Mg_17_Al_12_ phase.

As shown in Figure 8b,c, the original coarse β-phase was effectively crushed and evenly distributed within the α-Mg matrix after FSP when the traverse speed was either 30 mm/min or 60 mm/min. At higher magnification, it was noticed that the β-phase was distributed as fine particles in the α-Mg matrix, as depicted in Figure 8f. As depicted in Figure 8d,e, there was a noticeable increase in the clustering of the second-phase particles in the SZ when the traverse speed was increased from 90 mm/min to 120 mm/min. This can be attributed to the fact that there was not enough stirring time in the SZ at an excessively high traverse speed, resulting in inadequate fragmentation and dispersion of the coarse second phase.

### 3.3. Microhardness

**Figure 9:**
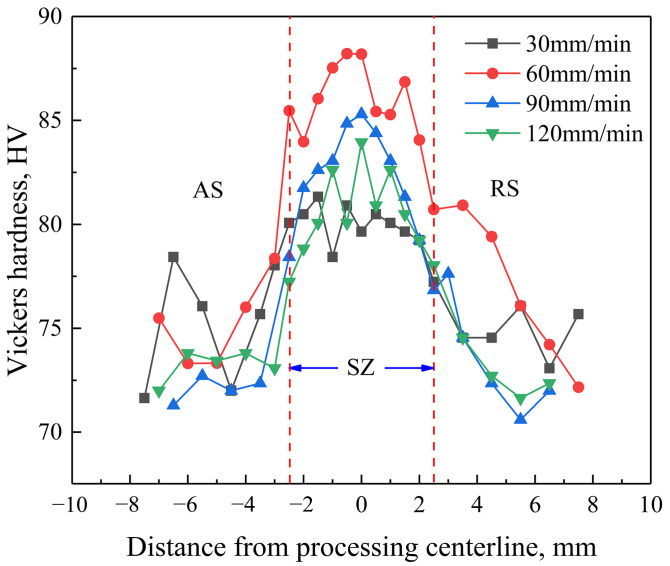
Microhardness distribution of the cladding layer after FSP.

Figure 9 displays the microhardness distribution of the processed cladding layer, and Table 4 presents the average grain sizes and average microhardness of the unprocessed cladding layer and the SZ. The average microhardness of the SZ was enhanced as a result of the combined influence of fine grain strengthening and second-phase dispersion strengthening caused by FSP, in comparison to the cladding layer prior to FSP. When the traverse speed increased from 30 mm/min to 60 mm/min, the average microhardness of the SZ increased as the average grain size decreased. Nevertheless, the average microhardness of the SZ dropped when the traverse speed was raised to 90 mm/min, although the average grain size further decreased. When the traverse speed was increased beyond a certain critical value, the second phase could not be adequately crushed, weakening the second-phase dispersion strengthening effect induced by FSP and, thus, leading to a decrease in the average microhardness of the SZ. When the traverse speed was increased to 120 mm/min, the stirring effect was further weakened, resulting in a continuous fall in the average microhardness. Furthermore, because the grain size increased with the increase in the distance to the processing center, the microhardness distribution of the processed cladding layer exhibited an inverted V-shape.

### 3.4. Tensile Properties

**Figure 10:**
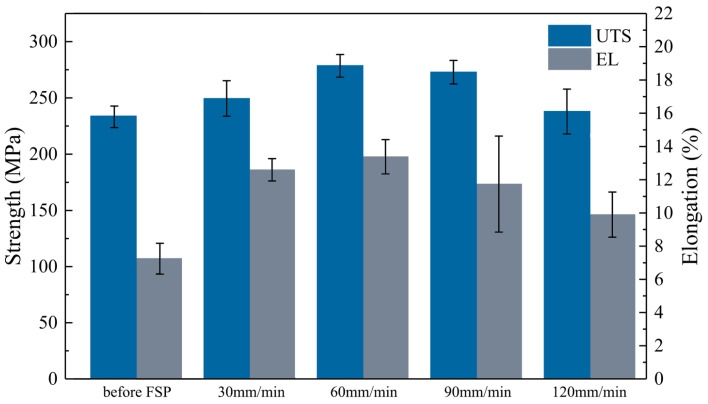
Tensile properties of the samples.

As shown in Figure 10, the unprocessed cladding layer had an average ultimate tensile strength (UTS) of 233.4 MPa and an average elongation of 5.6%; after FSP with different traverse speeds, the cladding layer’s elongation and average UTS were both improved. The combined impact of second-phase fragmentation and dispersion, together with grain refining, is responsible for this shift. As the traverse speed increased, there was an initial increase followed by a decrease in both the average UTS and elongation of the processed cladding layer. The average UTS and elongation both attained their maximum values at a traverse speed of 60 mm/min, i.e., 278.5 MPa and 13.4%, respectively.

**Figure 11:**
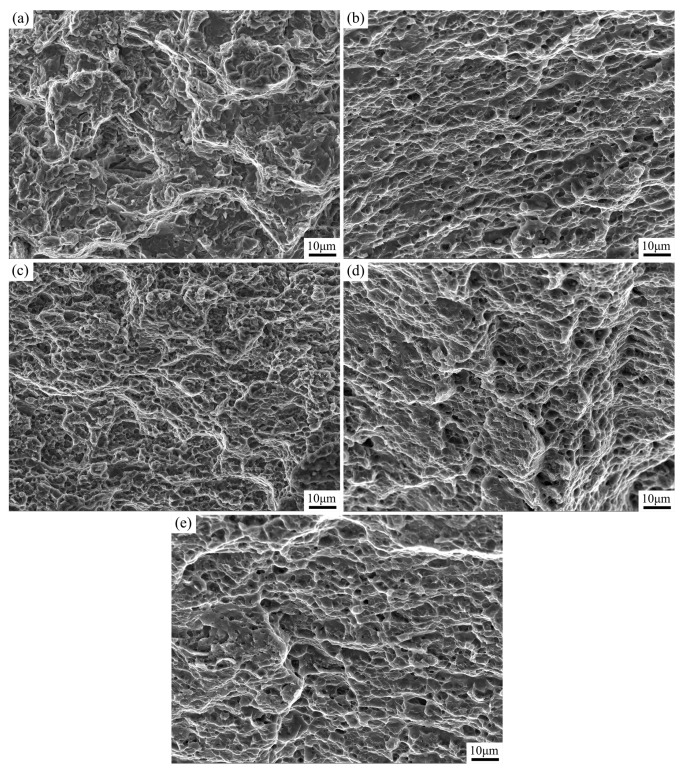
Fracture morphology of the samples: (**a**) before FSP; (**b**) 30 mm/min; (**c**) 60 mm/min; (**d**) 90 mm/min; and (**e**) 120 mm/min.

As shown in Figure 11a, the fracture surface of the unprocessed cladding layer exhibited typical brittle fracture characteristics with numerous tearing edges and no evident ductile dimples. However, when the traverse speeds were 30 mm/min, 60 mm/min, and 90 mm/min, the tensile fracture surface of the processed cladding layer displayed typical ductile fracture characteristics with a significant presence of dimples, as shown in Figure 11b–d. In addition, as the traverse speed increased, the dimple size gradually increased with a decrease in the dimple depth. At a traverse speed of 120 mm/min, the number of dimples on the fracture surface of the processed cladding layer significantly decreased, and a considerable number of tearing edges were present on the fracture surface. This indicates that the fracture mode of the processed cladding layer obtained using this parameter was a ductile–brittle facture with lower plasticity compared to the other processed cladding layer specimens.

## 4. Conclusions

In this study, the FSP was introduced to treat the AZ91 magnesium alloy cladding layers prepared using the CMT technique, and the influence of the traverse speed of the stirring head on the microstructure and mechanical characteristics of the cladding layers was investigated. The main conclusions obtained from this paper are as follows.

(1) Defects such as pores and cracks were formed in the cladding layer, and the FSP could effectively eliminate the defects existing in the cladding layer. When the rotation speed of the stirring head remained constant at 300 r/min, the intermediate width of the SZ decreased as the traverse speed increased.
(2) FSP-induced dynamic recrystallization could considerably reduce the average grain size of the cladding layer from 17.6 μm to 5.6 μm. Furthermore, the elongated β-Mg_17_Al_12_ second phases, which were originally present at the borders of the grains, were fragmented and scattered throughout the α-Mg matrix after FSP. As the traverse speed increased, the average grain size of the SZ initially declined and then rose.
(3) The synergistic impact of fine-grain strengthening and second-phase dispersion strengthening caused by FSP resulted in a significant improvement in average micro-hardness, UTS, and elongation of the cladding layer, and these indexes all exhibited an initial increase followed by a decrease as the traverse speed increased. The unprocessed cladding layer presented typical brittle fracture features, whereas a majority of the cladding layers after FSP exhibited ductile fracture characteristics.
(4) After FSP with a rotation speed of 300 r/min and a traverse speed of 60 mm/min, the CMT cladding layer of AZ91 magnesium alloy showed the best mechanical properties. The average microhardness, UTS, and elongation of the processed CMT cladding layer increased by 16.9%, 19.3%, and 139.3%, respectively, compared to those of the unprocessed cladding layer.

The results obtained in this study can provide a theoretical basis for improving the cladding layers of magnesium alloys, and it is beneficial to encourage the wider utilization of wire arc additive manufacturing (WAAM) and FSP hybrid technology for repairing damaged component surfaces made of magnesium alloy.

## Figures and Tables

**Table 1:** Chemical components of the materials (wt.%).

Materials	Al	Zn	Mn	Fe	Si	Ni	Cu	Mg
Substrate	8.70	0.58	0.24	0.0020	0.020	0.00100	0.0050	Bal.
Wire	8.99	0.65	0.26	0.0018	0.037	0.00043	0.0025	Bal.

**Table 2:** CMT cladding parameters.

Parameters	Value
Wire feed speed (m/min)	12
Traverse speed (m/min)	0.3
Flow rate of the shielding gas (L/min)	15
Inter-pass cooling time (s)	120

**Table 3:** FSP parameters.

Test Number	Rotation Speed (r/min)	Traverse Speed (mm/min)
1	300	30
2	300	60
3	300	90
4	300	120

**Table 4:** Average grain size and average microhardness of the samples.

Parameters	Average Grain Size (μm)	Average Microhardness (HV_0.1_)
before FSP	17.6	73.2
30 mm/min	6.2	80.0
60 mm/min	5.6	85.6
90 mm/min	3.4	82.8
120 mm/min	6.0	81.0

## Data Availability

Data available on request due to restrictions e.g., privacy or ethical.
